# Nutritional status of patients with phenylketonuria in Japan

**DOI:** 10.1016/j.ymgmr.2016.08.005

**Published:** 2016-08-20

**Authors:** Yoshiyuki Okano, Toshikazu Hattori, Hiroki Fujimoto, Kaori Noi, Miki Okamoto, Toshiaki Watanabe, Ryoko Watanabe, Rika Fujii, Tomoko Tamaoki

**Affiliations:** a Department of Genetics, Hyogo College of Medicine, Nishinomiya 663-8501, Japan; b Okano Children's Clinic, Izumi 594-0071, Japan; c Nutrition Dietary Section, Osaka City University Hospital, Osaka 545-0051, Japan; d Department of Health and Nutrition, Faculty of Human Science, Osaka Aoyama University, Mino 562-8580, Japan; e Department of Dietary Environment Analysis, School of Human Science and Environment, University of Hyogo, Himeji 670-0092, Japan; f The Graduate School of Comprehensive Rehabilitation, Osaka Prefecture University, Habikino 583-8555, Japan; g Department of Clinical Genetics, Hyogo College of Medicine, Nishinomiya 663-8501, Japan

**Keywords:** Phenylketonuria, Phenylalanine hydroxylase, Nutrition, Microelement, Vitamin, Selenium, Biotin, Magnesium, Zinc, Iodine, Phosphorus

## Abstract

Accumulating evidence suggests that hyperphenylalaninemia in phenylketonuria (PKU) can cause neuropsychological and psychosocial problems in diet-off adult patients, and that such symptoms improve after resumption of phenylalanine-restricted diet, indicating the need for lifetime low-phenylalanine diet. While limiting protein intake, dietary therapy should provide adequate daily intake of energy, carbohydrates, fat, vitamins, and microelements. We evaluated nutrient balance in 14 patients with classical PKU aged 4–38 years. Approximately 80–85% of the recommended dietary allowance (RDA) of protein in Japanese was supplied through phenylalanine-free (Phe-free) milk and Phe-free amino acid substitutes. Nutritional evaluation showed that the calorie and protein intakes were equivalent to the RDA. Phenylalanine intake was 9.8 ± 2.2 mg/kg of body weight/day, which maintained normal blood phenylalanine concentration by the 80% Phe-free protein rule. The protein, fat, and carbohydrate ratio was 9.5:23.9:66.6% with relative carbohydrate excess. Phe-free milk and amino acid substitutes provided 33.7% of carbohydrate, 82.1% of protein, and 66.7% of fat intake in all. Selenium and biotin intakes were 25.0% and 18.1% of the RDA and adequate intake (AI) for Japanese, respectively; both were not included in Phe-free milk. PKU patients showed low serum selenium, low urinary biotin, and high urinary 3-hydroxyisovaleric acid in this study. The intakes of magnesium, zinc, and iodine were low (71.5%, 79.5%, and 71.0% of the RDA, respectively) and that of phosphorus was 79.7% of the AI, although they were supplemented in Phe-free milk. PKU patients depend on Phe-free milk and substitutes for daily requirement of microelements and vitamins as well as protein and fat. Development of low-protein food makes it possible to achieve the aimed phenylalanine blood level, but this lowers the intake of microelements and vitamins from natural foods. The dietary habits vary continuously with age and environment in PKU patients. We recommend the addition of selenium and biotin to Phe-free milk in Japan and the need to review the composition of microelements and vitamins in A-1 and MP-11 preparations.

## Highlights

• 80- 85% of the recommended amount of protein should be supplied from Phe-free amino acid substitutes for good control.
• Nutritional evaluation in PKU patients is necessary for dietary control and for checking the contents of medical foods.
• Selenium, and biotin in Phe-free milk in Japan is insufficient and should be supplied immediately.
• Microelement from early children to adults is necessary more than in infants due to low protein diet.

## 1 Introduction

Phenylketonuria (PKU) is an autosomal recessive disorder caused by deficiency in hepatic phenylalanine hydroxylase and is usually diagnosed early in life. Unless the affected child is maintained on a strict low-phenylalanine diet, PKU leads to mental retardation, seizures, behavioral difficulties, and other neurological symptoms [1]. The introduction of newborn mass screening for this disorder allowed early diagnosis of PKU, increased the chance of normal development of affected infants through early-intervention dietary treatment, and markedly improved long-term prognosis. At first, restriction of phenylalanine intake was thought to be necessary for brain development in infants and children with PKU. The initial protocol did not state the low-phenylalanine diet prescribed for classical PKU in the second decade of life. However, accumulating evidence in the last several years suggested that hyperphenylalaninemia can cause neuropsychological and psychosocial problems in diet-off adults, and that such symptoms improved after resumption of phenylalanine-restricted diet [2], [3], [4], [5]. Thus, diet therapy is necessary and important over the lifetime of patients with PKU.

The dietary therapy for PKU includes restriction of intake of natural foods, such as high protein, but at the same provides protein at amounts similar to those consumed by age- and sex-matched healthy subjects, using phenylalanine-free (Phe-free) protein substitutes to maintain blood phenylalanine concentration within the therapeutic range. Furthermore, it is important that the daily intake includes the same amount of energy and balanced diet of carbohydrate, fat and protein, similar to sex- and age-matched healthy individuals. Therefore, Phe-free milk, A-1, MP-11, and low-protein rice, noodle, and bread are used for dietary therapy of PKU.

Phe-free milk, A-1 (a Phe-free amino acid substitute) and MP-11 (a low Phe peptide substitute) are available in Japan. The Phe-free milk is used with breast milk or general formula milk during the newborn period. It was approved for use as a medical product in 1980, and is supplied as a pharmaceutical product by Snow Brand Meg Milk Co., Ltd. (Tokyo). A-1 was developed by Snow Brand Meg Milk in 1981, and is comprised mainly of the same amino acids of Phe-free milk but does not contain fat, carbohydrates, microelements, or vitamins. On the other hand, MP-11 was developed in 1998 by Morinaga Milk Industry Co., Ltd. (Tokyo), and based on the nutritional requirements of PKU patients at that time; the product contains high concentrations of microelements but not vitamins [6]. MP-11 contains 280 mg of phenylalanine per 100 g. MP-11 consists of the amino acids dimers, and thus its digestion and absorption are better than the mixture of amino acid substitutes without the smell of amino acids. Patients can obtain A-1 and/or MP-11 through the Special Milk Safety Development Committee free of charge as social contribution from the companies and government subsidies.

Even if PKU patients have been detected in the newborn mass screening, patients with high blood phenylalanine concentration showed low IQ. Moreover, previous studies showed a negative correlation between blood phenylalanine concentration and IQ [7]. Magnetic resonance imaging (MRI) and electroencephalographic (EEG) findings indicate the need to maintain blood phenylalanine concentration below 480 μmol/L [8], [9]. Blood phenylalanine concentration (> 600 μmol/L) are reported to be associated with oxidative stress state in human [10]. Therefore, recent recommendations impose more strict limitation on phenylalanine intake in adults to maintain low phenylalanine concentration, compared with previous recommendations. At present, a threshold value of 600 μmol/L or less is set in many countries as the upper limit for blood phenylalanine concentration for patients aged ≥ 16 years [11]. The first Japanese guideline published in 1977 did not place a cap on blood phenylalanine concentration in adult patients with PKU. However, the second 1995 guidelines limited blood phenylalanine in such patients within the range of 180 to 900 μmol/L. On the other hand, the most recent Japanese guidelines in 2012 set the preferred blood phenylalanine concentration in adult PKU patients between 120 and 600 μmol/L.

Various types of low protein diet products (rice, noodles, bread) have been recently developed for patients with renal diseases. The development of low-protein rice (1/5–1/30 protein of normal rice) of good flavor has made it easier for PKU patients to maintain blood phenylalanine within the above range. This is particularly important for Japanese patients since boiled rice is the staple food in Japan. Strict limitation of consumption of natural protein may be associated with low intake of microelements and vitamins that are only present in natural protein. In addition, the Ministry of Health, Labour and Welfare has prohibited to supplement with biotin, selenium, and carnitine to formula milk including Phe-free milk and Phe-free amino acid substitutes. Biotin was permitted recently. Thus, the use of only these preparations could potentially lead to deficiency of microelements and vitamins. Thus far, there are hardly any reports on nutritional assessment of PKU patients in Japan, though zinc, selenium, iron, and vitamin B_12_ deficiency have been reported in PKU patients living in other countries [12], [13], [14], [15].

In the present report, we demonstrate that 80 to 85% of daily protein intake should be from Phe-free milk, A-1, and MP-11 in PKU patients. We evaluate the nutritional intake, including those of the three major nutrients, microelements, and vitamins, in Japanese patients with PKU. We also report preliminary results of serum selenium, serum biotin, urinary biotin, and urinary 3-hydroxyisovaleric acid (3HIA).

## 2 Methods

### 2.1 Subjects

Nutritional evaluation was conducted in 14 patients (nine males) with classical PKU aged 4 to 38 years. All patients showed > 1200 μmol/L of phenylalanine without dietary treatment and had severe phenotypic mutations in both alleles. In the dietary therapy of these PKU patients, 80–85% of age- and gender-specific recommended dietary allowance (RDA) for protein according to the Dietary Reference Intakes for Japanese 2010 [16] was taken from the Phe-free protein comprising Phe-free milk with/without Phe-free amino acid powder (A-1), and low phenylalanine peptide powder (MP-11). The natural protein taken is within 15–20% of the RDA. PKU patients generally consumed natural low-protein food and rice, noodle, and bread that have been improved to a low protein content. We have regulated the dietary food, protein restriction, and Phe-free materials in PKU patient according to the blood phenylalanine value and nutritional assessment. Table 1 lists the composition of Phe-free milk, A-1, and MP-11.

### 2.2 Nutritional evaluation

In the Dietary Reference Intakes for Japanese 2010, the “Estimated average requirement (EAR)” represents the estimated daily intake required by 50% of the population. Furthermore, the RDA means the estimated daily intake by 97–98% of the Japanese population, with a probability of lack of 2.5%. When the EAR and the RDA cannot be used based on scientific grounds, the “adequate intake (AI)” is set as the sufficient amount to maintain constant nutritional status, which is, in general, larger than the RDA. The “Tolerable upper intake level (UL)” is the excessive intake amount that could potentially cause health disturbances.

We evaluated food charts described by the patients, their parents, and/or caretakers from 2010 to 2014, and evaluated the three major nutrients, microelements, and vitamins, using the Standard Tables of Food Composition in Japan 2010 [17]. The average values from the 2–4 days food charts were considered for analysis. Each nutrient was calculated relative to that of gender- and age-specific RDA or AI amount. For nutritional evaluation, the subjects were divided in two groups; the Phe-free milk group (subjects who used Phe-free milk alone) and the A-1/MP-11 group (subjects who were provided with A-1/MP-11, including Phe-free milk for adequate protein supply) (Table 2). In patients who underwent several nutritional evaluations, we evaluated food charts at one year or more intervals. In each group, a maximum of only two nutritional evaluations were conducted per patient.

### 2.3 Measurement of phenylalanine, selenium, biotin, and 3-hydroxyisovaleric acid

The blood phenylalanine was measured quantitatively by tandem mass spectrometry (The TQ Detector of Waters Company) for newborn mass screening with non-derivatization method [18], set at the Multiple Reaction Monitoring mode. Labeled Amino Acid Standards SET A (NSK-A-OP) and Labeled Carnitine Standards SET B (NSK-B-OP) (Cambridge Isotope Laboratories Company) were used as the internal standards. Biotin contents, free and protein-binding biotin, in the serum and urine were determined separately using microtiter plate by the microbiological assay with *Lactobacillus plantarum* ATCC 8014, as described elsewhere [19]. Urinary 3HIA was determined using HPLC after derivatization with 2-nitrophenylhydrazine hydrochloride as described elsewhere [20]. Measurement of serum selenium level was outsourced to the LSI Medience Co. (Tokyo).

### 2.4 Statistical analysis

Values were expressed as mean ± SD. Differences between two groups were examined for statistical significance using the two-tailed Student's *t*-test. A P value < 0.05 was considered statistically significant.

## 3 Results

### 3.1 Patients

**Fig. 1:**
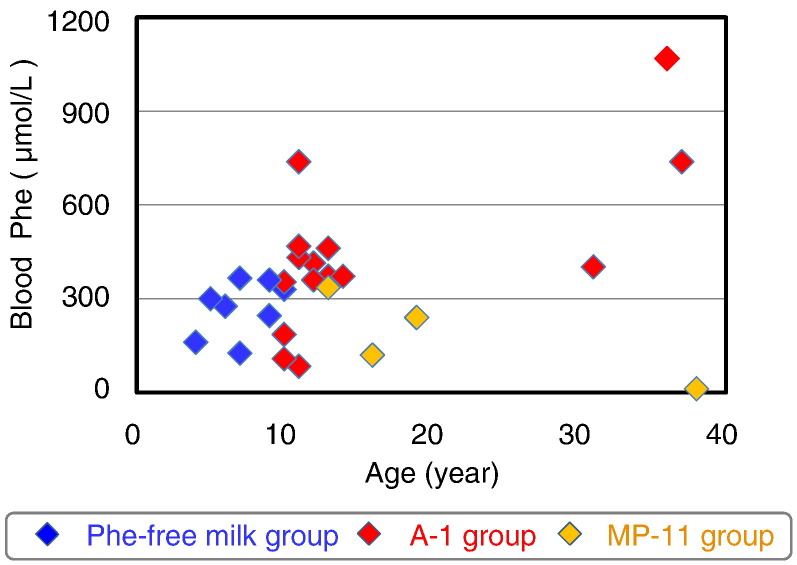
Blood phenylalanine concentration in PKU patients where Phe-free milk and amino acid substitutes account for 80–85% of protein intake.

Six PKU patients aged < 10 years could be treated with Phe-free milk alone. To secure sufficient daily protein intake, 12 patients aged > 10 years required A-1/MP-11 with Phe-free milk (Table 2). During the study period, three males and one female were switched from the Phe-free milk group to the A-1/MP-11 group for proper body growth. In the A-1/MP-11 group, A-1 was used in 10 patients, while MP-11 was used in 3 patients. One female patient was switched from A-1 to MP-11. The average intake of Phe-free milk was 200–250 g and this value was not significantly different between the two groups (Table 2). Fig. 1 shows blood phenylalanine concentration according to age and nutritional group. Blood phenylalanine concentration tended to increase with age, but was < 600 μmol/L in all except for a patient (Fig. 1).

### 3.2 Intake of energy and of three major food components

**Fig. 2:**
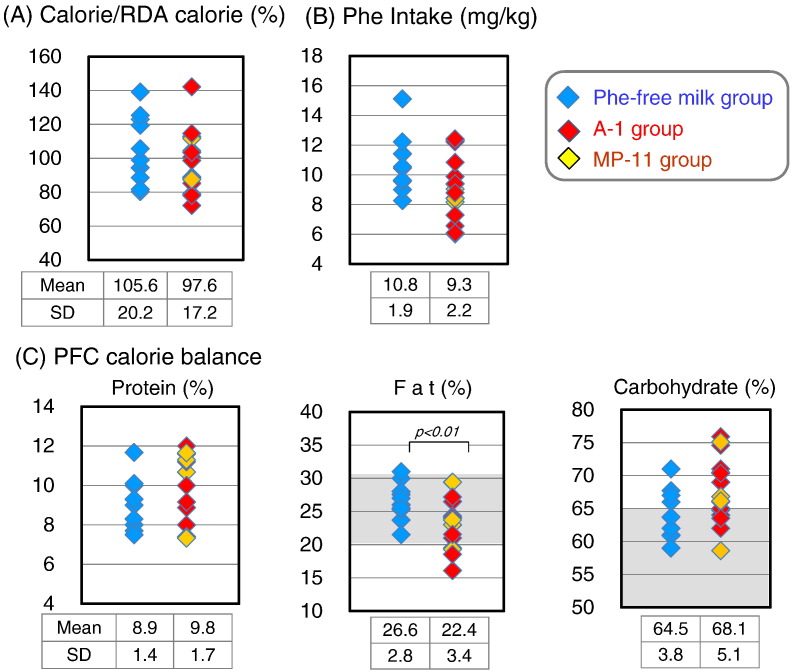
Status of calorie intake, protein intake, and calorie balance for protein, fat, and carbohydrates in the Phe-free milk group and A-1/MP-11 group. (A) Ratio of calorie intake to age-and gender-specific RDA. (B) Average phenylalanine intake per body weight per day. (C) Calorie balance according to protein, fat, and carbohydrates. Data are mean ± SD. P values by Student's *t*-test.

We investigated the gender- and age-specific energy requirements (intermediate physical activity level) and the nutritional balance of protein, lipid, and carbohydrates (Fig. 2). The average energy intake in both of Phe-free milk and A-1/MP-11 groups was 100.6 ± 18.4% of the RDA, which was approximately equivalent to that of healthy individuals. There was also no significant difference in energy intake between Phe-free milk group (105.6 ± 20.2%) and A-1/MP-11 group (97.6 ± 17.2%). About half of the energy intake was obtained from Phe-free milk with/without A-1/MP-11 (49.4 ± 8.9%). The protein, fat, and carbohydrates calorie (PFC) ratio was 9.5%: 23.9%: 66.6% for all patients, indicating slightly excessive carbohydrate intake (Fig. 2C). The percentages of protein and carbohydrate calories were not significantly different between the Phe-free milk and A-1/MP-11 groups. However, fat calorie in PFC ratio was significantly higher in the Phe-free milk group (26.6%) than the A-1/MP-11 group (22.4%). A lot of the fat in the PKU patients is supplied from Phe-free milk. With regard to the source of each food component, 37.7 ± 10.2% of carbohydrates, 66.7 ± 9.7% of fat, and 82.1 ± 5.5% of protein were obtained from the Phe-free milk and A-1/MP-11.

**Fig. 3:**
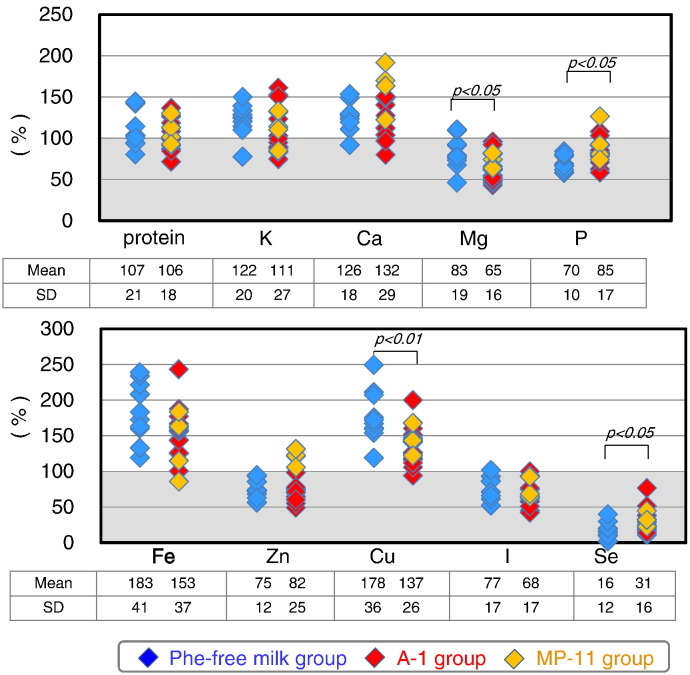
Intake of microelements in PKU patients relative to age-and gender-specific RDA or AI in the Phe-free milk and A-1/MP-11 groups. Data are mean ± SD. P values by Student's *t*-test.

The protein intake was 106.4 ± 18.7% of the RDA, and was approximately equivalent to that in healthy individuals. There was no significant difference in protein intake between the Phe-free milk group (107.1 ± 21.0%) and A-1/MP-11 group (106.1 ± 17.9%) (Fig. 3). Analysis of protein intake in the Phe-free milk group indicated that 17.4 ± 4.2% of such protein was natural protein while 82.6 ± 4.2% was from Phe-free milk. In comparison, 16.7 ± 6.7% was from natural protein, 22.1 ± 9.2% from A-1/MP-11, and 61.2 ± 8.1% from Phe-free milk in the A-1/MP-11 group. The intake of natural protein as a percentage of total protein was < 20% in both groups.

Phenylalanine intake in the Phe-free milk group (10.8 ± 1.9 mg/kg body weight/day) was comparable to that of the A-1/MP-11 group (9.3 ± 2.2 mg/kg/day), and the average of the entire group was 9.8 ± 2.2 mg/kg/day, indicating good control (Fig. 2).

### 3.3 Microelements and vitamins

Selenium intake was significantly lower in PKU patients (25.3 ± 16.2% of the RDA, Fig. 3). Furthermore, the intake levels of phosphorus (79.7 ± 16.0% of the AI), magnesium (71.5 ± 19.0% of the RDA), zinc (79.5 ± 21.0% of the RDA), and iodine (71.0 ± 17.5% of the RDA) were significantly lower in PKU patients. The respective intake levels of magnesium, zinc, and iodine were also lower 84.1 ± 22.3%, 93.5 ± 24.7%, and 95.6 ± 22.2% of the EAR. The intake levels of magnesium and copper were significantly higher in the Phe-free milk group (age, ≤ 10 year) compared with the A-1/MP-11 group (age, ≥ 10 years), since the RDA levels of magnesium and copper in 5-year-old children are three times lower than those of adults.

**Fig. 4:**
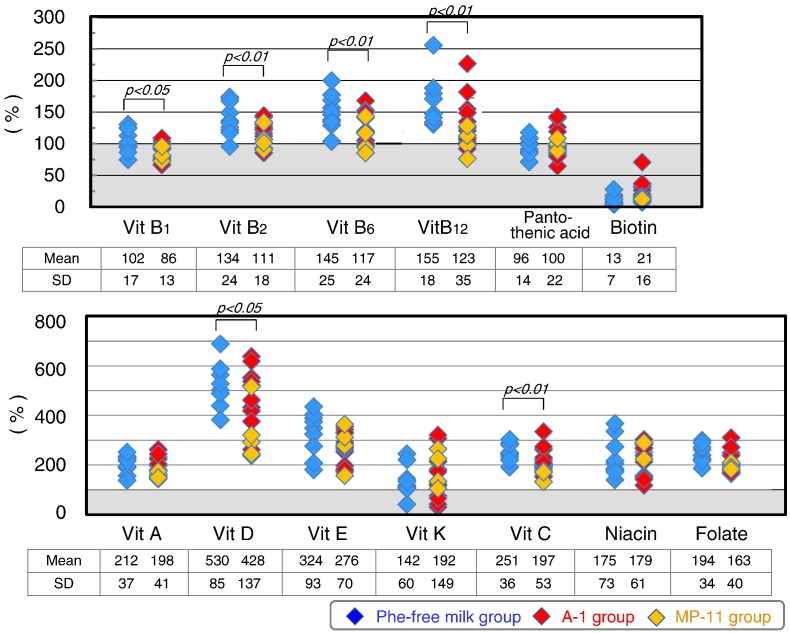
Vitamin intake in PKU patients relative to age-and gender-specific RDA or AI in the Phe-free milk and A-1/MP-11 groups. Data are mean ± SD. P values by Student's *t*-test.

Biotin intake was low in PKU patients (18.1 ± 13.5% of the AI) (Fig. 4), mainly because this vitamin was not a component of the Phe-free milk. The intake levels of the other tested vitamins in PKU patients were generally close to those of the RDA and AI, and were not beyond the upper limits in any of the patients. The intake levels of vitamin B group, vitamin C, and vitamin D were significantly higher in the Phe-free milk group compared to the A-1/MP-11 group. The Phe-free milk contains high supplements of vitamins. Phe-free milk intake was higher in the Phe-free milk group than A-1/MP-11 group due to differences in body physique. Biotin intake level was not significantly different between the Phe-free milk group and A-1/MP-11 group.

**Fig. 5:**
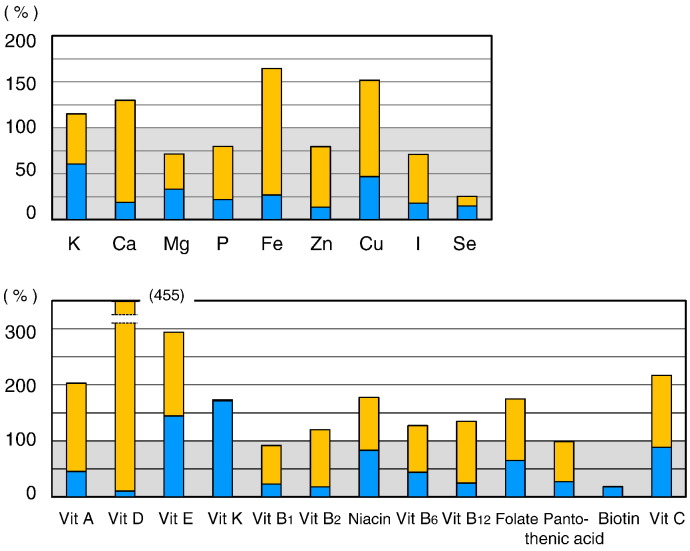
Percentages of intake of microelements and vitamins from natural foods and Phe-free milk, A-1, and MP-11 relative to age-and gender-specific RDA or AI. *Blue (Lower) bars:* percentage intake from natural foods relative to the RDA or AI. *Orange (Upper) bars:* percentage of intake from Phe-free milk, A-1, and MP-11 relative to the RDA or AI. (For interpretation of the references to colour in this figure legend, the reader is referred to the web version of this article.)

Fig. 5 shows the relative levels of microelements and vitamins. The intake levels of the majority of microelements in natural foods were < 50% of the RDA or AI. The sources of these microelements were mainly the Phe-free milk and MP-11. The total intake of selenium was only 25.3% of the RDA. Selenium intake from natural foods represented only 14.3% of the RDA.

Any vitamin K or biotin has not been added to Phe-free milk. Therefore, biotin intake came from natural foods and only 18% of the AI. On the other hand, vitamin K intake in natural foods amounted to 170% of the RDA. Except for vitamin E, vitamin K, niacin, and vitamin C, which are available in natural foods, the intake of other vitamins present in natural foods was inadequate in PKU patients. The most important source of vitamins was Phe-free milk.

### 3.4 Improvement of microelements and vitamins

**Fig. 6:**
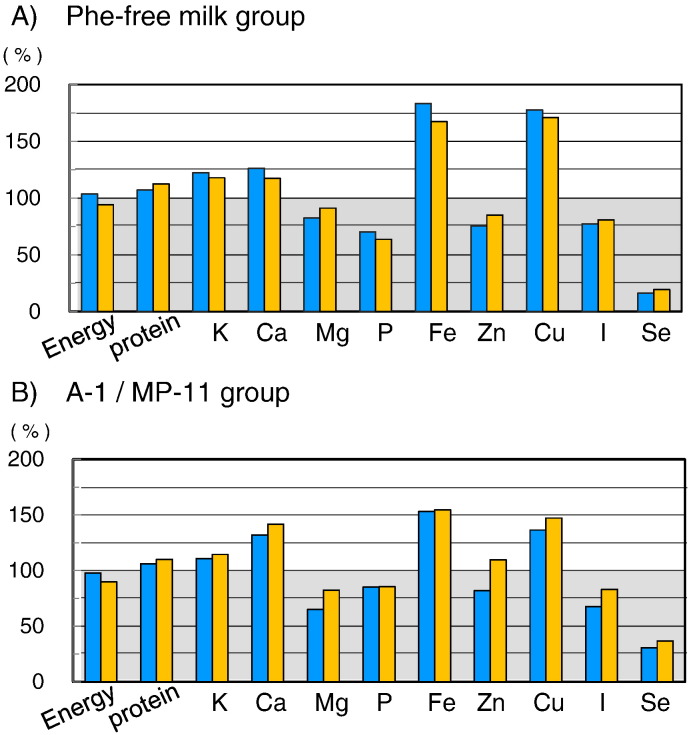
Estimated changes in energy, protein, and microelements intakes by the simulation to replace 20% protein in the Phe-free milk with MP-11in Phe-free milk group and A-1/MP-11 group. In Phe-free milk group, the source of protein intake was adjusted to be 20% from natural protein, 20% from MP-11, and 60% from Phe-free milk. In the A-1/MP-11 group, the source of protein intake was adjusted to be 20% from natural protein, 40% from MP-11, and 40% from Phe-free milk. T *Blue (left side) bars:* before estimation, *orange (right side) bar:* after estimation. (For interpretation of the references to colour in this figure legend, the reader is referred to the web version of this article.)

MP-11 contained various micronutrients, such as calcium, magnesium, iron, zinc, copper, but not biotin or selenium (Table 1). While the use of MP-11 increased zinc intake (Fig. 3), it did not improve magnesium, phosphorus, and iodine intake levels. Therefore, we examined whether it was possible to improve the intake of microelements by increasing the intake of MP-11. In the Phe-free milk group, we estimated that 20% of the RDA of protein was obtained from MP-11, and that the same amount of protein should be reduced from Phe-free milk. In other words, the source of protein intake was adjusted to be 20% from natural protein, 20% from MP-11, and 60% from Phe-free milk. In the A-1/MP-11 group, we estimated that 40% of the RDA of protein was obtained from MP-11. In other words, the source of protein intake was adjusted to be 20% from natural protein, 40% from MP-11, and 40% from Phe-free milk. These adjustments increased zinc intake, and the intake became approximately equivalent to the RDA of zinc in the A-1/MP-11 group (Fig. 6). However, increase in the intake of MP-11 did not improve the intake of magnesium, phosphorus, or iodine in both the Phe-free milk group and the A-1/MP-11 group, and zinc in the Phe-free milk group. These results indicated that we cannot expect improvement in microelements intake by increasing the amount of the MP-11, rather, improvement in microelement contents of MP-11 is required.

#### 3.5 Preliminary results of selenium, biotin, and 3-hydroxyisovaleric acid (Fig. 7)

The serum selenium of all 11 PKU patients were less than the reference value (10.6–17.4 μg/dL) and the average decreased to 5.66 ± 2.12 μg/dL. The lack of the selenium intake conducted to a decrease in serum selenium. The total biotin and free biotin did not decrease in the blood samples. However, urinary free biotin in 6 out of 7 patients were less than the reference value (4.4–25 μmol/mol creat). The mean value also reduced to 1.48 ± 1.66 μmol/mol creat. Urinary 3HIA increased in all 6 patients. The insufficient intake of biotin did not affect biotin in serum, but resulted in the decrease of urinary biotin, and the increase of urinary 3HIA.

## 4 Discussion

Dietary regulation in PKU is a delicate and difficult therapy. Since food is strongly restricted, compliance is a major factor that determines blood phenylalanine concentration. The basic concept and protocol used for dietary treatment of PKU are common across the world. However, since each country, area, and individuals have different dietary habits, it is necessary to build dietary treatment protocol according to the lifestyle. The PFC balance for Japanese is 13% protein, 29% fat, and 58% carbohydrates; whereas the PFC balance for European and American adults is 13% protein, 36–42% fat, and 45–50% carbohydrates. Thus, the Japanese diet is low fat high carbohydrate food compared with the Western diet [21]. In other words, the intake of meat, milk, oils, and fats is less in Japan than in the US and European countries. These differences in food could explain why protein restriction is well tolerated by Japanese PKU patients.

With respect to dietary therapy, we determined the need of Phe-free protein for classical PKU, which generally provides 80–85% of the RDA for protein. Next, we evaluated and adjusted the restriction on dietary contents of protein from natural foods, which form 20% of the RDA for protein. This protein amount can introduce to phenylalanine intake of 10–16 mg/kg/day in all ages since early childhood. And this phenylalanine intake satisfies 10–20 mg/kg/day which can maintain blood phenylalanine in < 600 μmol/L in classical PKU patients [22]. Actually, patients were able to achieve the target blood phenylalanine concentration. At the same time, we demonstrated that energy need of PKU patients was similar to that of age- and gender-matched healthy individuals, and that about 50% of energy requirement in PKU patients was from Phe-free milk and A-1/MP-11. With regard to the source of nutrients, natural food included 62% carbohydrates, 33% fat and 18% protein. In other words, Phe-free milk does not only supplement phenylalanine-free protein, but also supplements fat.

Biotin is an essential water soluble vitamin that cannot be synthesized in humans, and the required amount is maintained by both dietary intake from foods and synthesis from intestinal bacteria. Biotin mostly couples with protein in food. Biotin is present in the liver, egg yolk, soybean, and grains, and in small amounts in vegetables and fruits [23], [24], [25]. In PKU patients, the intake of protein is strictly limited and the intake of biotin supplied with protein is also greatly reduced. Phe-free milk and A-1 do not contain biotin, while MP-11 contains a tiny amount of biotin (0.6 μg/100 g). Nutritional evaluation showed that biotin intake was very low (18.1 ± 13.5% of the AI). Then, insufficient biotin intake in PKU patients did not provide a decrease in serum total and free biotin, but a decrease in urinary free biotin and an increase in urinary 3HIA. This increased urinary excretion of 3HIA reflects decreased activity of biotin-dependent enzyme; 3-methylcrotonil-CoA carboxylase, meaning the lack of biotin in the diet including Phe-free milk. The decrease of urinary free biotin and the increase of urinary 3HIA were observed to be in similar level, both of which are sensitive indicators for the lack of biotin. Watanabe et al. [26], [27] also reported that the serum total and free biotin levels in PKU Japanese patients of all age were similar to those of normal subjects. Their results showed significantly lower urinary biotin levels in infants with PKU compared with control infants. Interestingly, urinary biotin levels in young children and adults with PKU were lower than those of the control and infants with PKU. This difference is due to the fact that infant with PKU uses both biotin-free Phe-free milk and biotin-containing breast milk/general formula milk and that young children and adults with PKU are mainly on Phe-free milk, A-1, MP-11, and low-protein foods that contain only small amounts of biotin. Thus, Japanese young children and adults with PKU appear to be on low intake of biotin from food and show a lack of biotin.

Biotin deficiency is associated with lack of appetite, dry skin, erosion, hair loss, ataxia, muscular hypotonia, and developmental delays [23], [24], [25]. In Japan, skin abnormalities and hair loss are reported to be due to low contents of biotin in special formulas for inborn metabolic disease and milk allergy [28], [29]. In this regard, administration of biotin improved skin lesions found in PKU patients (personal communication). Biotin deficiency is also related to teratogenicity in animal studies [30], [31]. Female PKU patients planning pregnancy are often placed on limited protein intake to prevent maternal PKU, which can result in severe biotin deficiency. The biotin should be supplemented in Phe-free milk.

Selenium is an essential microelement and is present in fish, meat, cereals, and egg. Selenium insufficiency has not been reported in Japanese on normal diet. However, low blood selenium levels have been reported in PKU patients and selenium deficiency has been reported in patients on long-term intravenous nutrition and enteral feeding [32], [33], [34], [35], [36], [37], [38], [39]. In Japan, Phe-free milk, A-1 and MP-11 contain small amounts of selenium; 1 μg, 2 μg, 11 μg/100 g, respectively. Our nutritional evaluation showed low selenium intake of 25.3 ± 16.2% of the RDA. Selenium intake in the Phe-free milk group was significantly lower than that of the A-1/MP-11 group (Fig. 3). This is probably due to intake of fewer selenium-containing natural foods by PKU patients of Phe-free milk group (children aged 4–10 years), compared with the A-1/MP-11 group. Actually, the serum selenium in classical PKU patients largely decreased. Owada et al. [6] also reported low blood selenium levels in Japanese PKU patients.

The clinical features of selenium deficiency vary widely and include those related to immunodeficiency, gastrointestinal dysfunction, hair loss, diarrhea, fatigue, garlic breath, sloughing off of nails, heart failure and pulmonary edema. These features are rare in patients with PKU [14], [40]. Low selenium intake and resultant low selenium blood levels are associated with hypothyroidism through a decrease in iodothyronine deiodination enzyme and oxidative stress status through a decrease in glutathione peroxidase [41], [42], [43]. Evaluation of various oxidative stress markers suggested a state of oxidative stress in PKU patients [10], [44]. Furthermore, selenium supplementation with recovery of glutathione peroxidase activity resulted in improvement of oxidative stress markers in PKU patients [45], [46]. Strong restriction of protein intake in pregnant PKU patients to maintain low phenylalanine blood level could lead to severe hyposelenemia [39]. To our knowledge, there are no reports of PKU patients with selenium deficiency in Japan. However, we recommend selenium supplementation in Phe-free milk to prevent selenium deficiency in patients with PKU.

In the present study, magnesium, phosphorus, zinc, and iodine intakes were < 80% of the RDA/AI and did not attain the EAR. These levels should be worrisome. Most microelements such as magnesium, phosphorus, zinc, and iodine are present in protein-rich food. These low levels are probably due to the limited intake of natural protein in PKU patients on diet therapy. The amount of most microelements in natural foods is < 50% and thus PKU patients greatly depend on Phe-free milk and amino acid substitutes. While vitamins are also added at sufficient concentrations to Phe-free milk, with the exception of biotin, microelements should also be added in sufficient amount to Phe-free milk and A-1/MP-11.

The nutrients of Phe-free milk are added with the assumption that it is being used with breast milk or as a general formula milk for neonates and infants. Therefore, Phe-free milk alone is not nutritious to be used for diet therapy of PKU patients after the neonatal weaning age to adulthood. Reliance on Phe-free milk alone is probably the reason for iron, copper, zinc, manganese, serine, vitamin B6, and B12 deficiencies reported in PKU patients [47], [48], [49], [50]. Phe-free amino acid substitutes for young children and adults other than Phe-free milk are available in European and American countries. These preparations contain various microelement and vitamin supplements based on the diet requirements of each age group [51].

MP-11 is a low-phenylalanine peptide powder used as a supplement to Phe-free peptide and microelements (calcium, magnesium, iron, copper, zinc, and iodine) in Japan. We simulated that MP-11 provided 20% and 40% of RDA of protein in the Phe-free milk group and the A-1/MP-11 group, respectively. Based on the above property, the use of MP-11 in large amounts would not improve the lack of microelements (magnesium, phosphorus, iodine, zinc). MP-11 was prepared based on nutritional evaluation of PKU patients during development, which target blood phenylalanine concentration was < 900 μmol/L in adults. It has recently changed to 600 μmol/L. The development of low-protein food resulted in limited use of natural protein, with subsequent reduction in the intake of microelements from natural foods and deficiency in microelement levels in the circulation even in those patients using MP-11. It is necessary to increase the concentrations of magnesium, phosphorus, iodine, and zinc in MP-11. To prevent low blood levels of microelements, MP-11 should be recommended for Japanese PKU patients from young child to adulthood.

In the dietary therapy of PKU, the target level of blood phenylalanine has been set lower based on accumulation of data from clinical studies. Furthermore, the development of Phe-free amino acid substitutes and low-protein food allowed the achievement of the targeted value of blood phenylalanine. In this regard, diet and eating habits in each country change, including the diet of PKU patients. In other words, the eating habits of PKU patients change with age and time, and are not rigidly constant. It is necessary to check the diet contents of PKU patients and conduct nutritional evaluation at various ages. The results of such evaluations should be incorporated in the diet therapy of individual PKU patients, which must also be designed to include Phe-free milk, A-1, and MP-11. It is most important that PKU patients should take in restricted phenylalanine and the same amount of energy and micronutrients and balanced diet of carbohydrate, fat and protein, similar to those of healthy individuals. In other words, PKU patients should be in a condition to intake the sufficient amount of RDA and AI in all nutrients. Now, we are not able to provide enough nutritional contents and quantity for PKU patients as those of healthy individuals using Japanese phenylalanine-free milk, A-1, and MP-11. Importantly, selenium and biotin deficiency is in a serious condition and these food elements should be added in Phe-free milk immediately or should be provided as a supplement. It is also necessary to supplement MP-11 and A-1 with sufficient concentrations of magnesium, phosphorus, iodine, and zinc.

## Figures and Tables

**Fig. 7:**
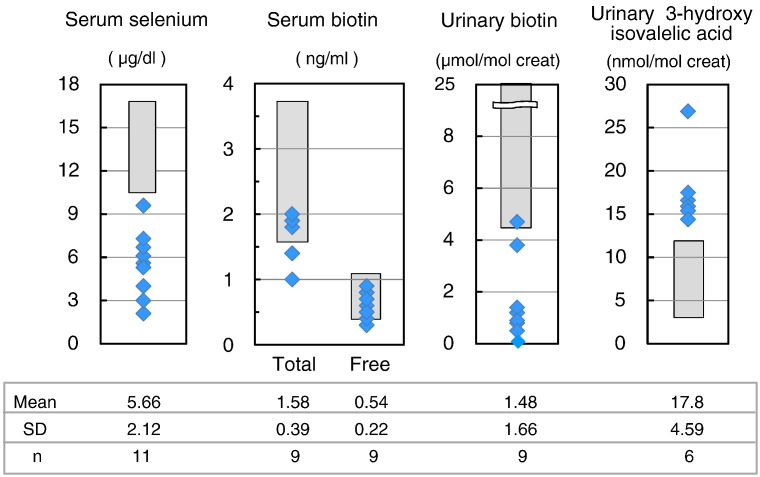
The distribution of serum selenium, serum free and total biotin, urinary free biotin, and urinary 3-hydroxyisovaleric acid levels in classical PKU patients. Data are mean ± SD. *n*: number of examined patients. *Dark box:* reference values.

**Table 1:** Nutritive composition of Phe-free milk and medical amino acid substitutions for PKU patients in Japan.

	Phe-free milk	Phe-free amino acid powder (A-1)	Low Phe peptide powder (MP-11)
Protein (g)	15.8	93.7	75
Fat (g)	17.1	0	0
Carbohydrate (g)	60.4	0	7.2
Ash content (g)	3.68	2.9	7.4
Water substance (g)	2.97	3.4	2.8
Energy (kcal)	458	375	329
Phe (mg)	0	0	280
Ca (mg)	360	0	1100
Mg (mg)	34	0	300
Na (mg)	168	880	620
K (mg)	440	0	1400
P (mg)	270	0	600
Cl (mg)	320	1900	300
Fe (mg)	6	0	15
Zn (mg)	2.5	0	20
Cu (μg)	280	0	1000
I (μg)	25	0	150
Se (μg)	1	2	11
Vit A (IU)	1500	0	0
Vit B1 (mg)	0.36	0	0
Vit B2 (mg)	0.6	0	0
Vit B6 (mg)	0.4	0	0
Vit B12	1	0	0
Vit C (mg)	48	0	0
Vit D (IU)	300	0	0
Vit E (mg)	4.38	0	0
Ca pantothenate (mg)	2	0	0
Niacin (mg)	5	0	0
Folate (mg)	0.1	0	0
Choline (mg)	50	0	0
Biotin (μg)	0	0	0.6

**Table 2:** Background of PKU patients and intake of Phe-free milk and A-1/MP-11 in the nutritional evaluation.

	Phe-free milk group	A-1/MP-11 group	Total
Number of patients	6 (males: 5)	12 (males: 7)	14 (males: 9)
Number of nutritional evaluation	10	17	27
Age (years)	4–10	10–38	4–38
A-1 (g)*	0	10 patients (12 ± 6, 6–27)	
MP-11 (g)*	0	3 patients (24 ± 6,16–28)	
Phe-free milk (g)*	203 ± 33 (130–240)	215 ± 36 (180–300)	

* Intake of amino acid substitutes (mean ± SD, min-max)

## References

[1] Scriver C.R., Kaufman S., Scriver C.R., Beaudet A.L., Sly W.S., Sly D. (2001). Hyperphenylalaninemia: phenylalanine hydroxylase deficiency. The Metabolic and Molecular Bases of Inherited Disease.

[2] Hanley W.B. (2004). Adult phenylketonuria. Am. J. Med..

[3] Lee P.J., Amos A., Robertson L., Fitzgerald B., Hoskin R., Lilburn M., Weetch E., Murphy G. (2009). Adults with late diagnosed PKU and severe challenging behaviour: a randomised placebo-controlled trial of a phenylalanine-restricted diet. J. Neurol. Neurosurg. Psychiatry.

[4] Trefz F., Maillot F., Motzfeldt K., Schwarz M. (2011). Adult phenylketonuria outcome and management. Mol. Genet. Metab..

[5] ten Hoedt A.E., de Sonneville L.M., Francois B., ter Horst N.M., Janssen M.C., Rubio-Gozalbo M.E., Wijburg F.A., Hollak C.E., Bosch A.M. (2011). High phenylalanine levels directly affect mood and sustained attention in adults with phenylketonuria: a randomised, double-blind, placebo-controlled, crossover trial. J. Inherit. Metab. Dis..

[6] Owada M., Aoki K., Yamaguchi S., Ohura T., Sawada Y., Takayanagi M., Higashi A., Kajikawa M., Hayasaka H., Matsuda I., Tada K., Oura T., Kitagawa T. (2000). Dietary treatment of phenylketonuria with low-phenylalanine peptide fortified with minerals and trace elements. J. Jpn. Pediatr. Soc..

[7] Owada M. (2009). Long term outcome of the patients with amino acid disorders detected by the neonatal screening program in Japan. Jpn. J. Mass Screening.

[8] Izumi M., Yamazaki H., Owada M. (2004). EEG pattern in phenylketonuria. J. Jpn. Pediatr. Soc..

[9] Kono K., Okano Y., Nakayama K., Hase Y., Minamikawa S., Ozawa N., Yokote H., Inoue Y. (2005). Diffusion-weighted MR imaging in patients with phenylketonuria: relationship between serum phenylalanine levels and ADC values in cerebral white matter. Radiology.

[10] Sanayama Y., Nagasaka H., Takayanagi M., Ohura T., Sakamoto O., Ito T., Ishige-Wada M., Usui H., Yoshino M., Ohtake A., Yorifuji T., Tsukahara H., Hirayama S., Miida T., Fukui M., Okano Y. (2011). Experimental evidence that phenylalanine is strongly associated to oxidative stress in adolescents and adults with phenylketonuria. Mol. Genet. Metab..

[11] Blau N., van Spronsen F.J., Levy H. (2010). Phenylketonuria. Lancet.

[12] Hanley W.B., Feigenbaum A., Clarke J.T., Schoonheyt W., Austin V. (1993). Vitamin B12 deficiency in adolescents and young adults with phenylketonuria. Lancet.

[13] Lee P., Smith I., Piesowicz A., Brenton D. (1999). Spastic paraparesis after anaesthesia. Lancet.

[14] Gassió R., Artuch R., Vilaseca M.A., Fusté E., Colome R., Campistol J. (2008). Cognitive functions and the antioxidant system in phenylketonuric patients. Neuropsychology.

[15] Barretto J.R., Silva L.R., Leite M.E., Boa-Sorte N., Pimentel H., Purificacao A.C., Carvalho G., Fontes M.I., Amorim T. (2008). Poor zinc and selenium status in phenylketonuric children and adolescents in Brazil. Nutr. Res..

[16] Kasuga M. (2010). Dietary Reference Intakes for Japanese in 2010 by the Ministry for Health, Labour and Welfare in Japan. http://www.mhlw.go.jp/bunya/kenkou/sessyu-kijun.html.

[17] Suzuki A. (2010). Standard Tables of Food Composition in Japan (2010) by Science Council Resource Survey Subcommittee, Ministry of Education, Culture, Sports, Science and Technology, National Gazette Sale Cooperative, Tokyo.

[18] Shigematsu Y., Hata I. (2009). Tandem mass spectrometric analysis of dried blood spot extracts without derivatization for newborn screening. Jpn. J. Mass Screening.

[19] Watanabe T., Yasumura S., Shibata H., Fukui T. (1998). Biotin status and its correlation with other biochemical parameters in the elderly people of Japan. J. Am. Coll. Nutr..

[20] Watanabe T., Oguchi K., Ebara S., Fukui T. (2005). Measurement of 3-hydroxyisovaleric acid in urine of biotin-deficient infants and mice by HPLC. J. Nutr..

[21] Kumakura I. (2012). Japanese Food Culture Text by Ministry of Agriculture, Forestry and Fisheries in Japan. http://www.maff.go.jp/j/keikaku/syokubunka/culture/eiyo.html.

[22] Güttler F. (1980). Hyperphenylalaninemia: diagnosis and classification of the various types of phenylalanine hydroxylase deficiency in childhood. Acta Paediatr. Scand. Suppl..

[23] Said H.M. (2012). Biotin: biochemical, physiological and clinical aspects. Subcell. Biochem..

[24] McMahon R.J. (2002). Biotin in metabolism and molecular biology. Ann. Rev. Nutr..

[25] Zempleni J., Kuroishi T. (2012). Biotin. Adv. Nutr..

[26] Watanabe T., Fukui T. (1998). Low biotin content of infant formulas made in Japan. Food Addit. Contam..

[27] Watanabe T., Yonekubo A., Kuwata T., Yamaguchi S., Kobayashi A., Yoshida I., Okano Y., Ohura T., Ohwada M., Matsuda L., Tada K., Oura T., Aoki K., Kitagawa T., Fukui T. (2005). Nutritional state of biotin in infants fed formulas and maternal milk. Vitamin.

[28] Hayashi H., Tokuriki S., Okuno T., Shigematsu Y., Yasushi A., Matsuyama G., Sawada K., Ohshima Y. (2014). Biotin and carnitine deficiency due to hypoallergenic formula nutrition in infants with milk allergy. Pediatr. Int..

[29] Fujimoto W., Inaoki M., Fukui T., Inoue Y., Kuhara T. (2005). Biotin deficiency in an infant fed with amino acid formula. J. Dermatol..

[30] Zempleni J., Teixeira D.C., Kuroishi T., Cordonier E.L., Baier S. (2012). Biotin requirements for DNA damage prevention. Mutat. Res..

[31] Mock D.M. (2005). Marginal biotin deficiency is teratogenic in mice and perhaps humans: a review of biotin deficiency during human pregnancy and effects of biotin deficiency on gene expression and enzyme activities in mouse dam and fetus. J. Nutr. Biochem..

[32] Litov R.E., Combs G.F. (1991). Selenium in pediatric nutrition. Pediatrics.

[33] Lombeck I., Kasperek K., Feinendegen L.E., Bremer H.J. (1975). Serum-selenium concentrations in patients with maple-syrup-urine disease and phenylketonuria under diet therapy. Clin. Chim. Acta.

[34] Longhi R., Rottoli A., Vittorelli A., Zecchini G., Bonabitacola T., Bertassi F., Riva E., Giovannini M. (1987). Trace elements nutriture in hyperphenylalaninemic patients. Long term follow up study. Eur. J. Pediatr..

[35] Reilly C., Barrett J.E., Patterson C.M., Tinggi U., Latham S.L., Marrinan A. (1990). Trace element nutrition status and dietary intake of children with phenylketonuria. Am. J. Clin. Nutr..

[36] Darling G., Mathias P., O'Regan M., Naughten E. (1992). Serum selenium levels in individuals on PKU diets. J. Inherit. Metab. Dis..

[37] Masumoto K., Nagata K., Higashi M., Nakatsuji T., Uesugi T., Takahashi Y., Nishimoto Y., Kitajima J., Hikino S., Hara T., Nakashima K., Nakashima K., Oishi R., Taguchi T. (2007). Clinical features of selenium deficiency in infants receiving long-term nutritional support. Nutrition.

[38] Procházková D., JarkovskýJ J., Vinohradská H., Konečná P., Machačová L., Doležel Z. (2013). Controlled diet in phenylketonuria and hyperphenylalaninemia may cause serum selenium deficiency in adult patients: the Czech experience. Biol. Trace Elem. Res..

[39] Lombeck I., Jochum F., Terwolbeck K. (1996). Selenium status in infants and children with phenylketonuria and in maternal phenylketonuria. Eur. J. Pediatr..

[40] Greeves L.G., Carson D.J., Craig B.G., McMaster D. (1990). Potentially life-threatening cardiac dysrhythmia in a child with selenium deficiency and phenylketonuria. Acta Paediatr. Scand..

[41] Wilke B.C., Vidailhet M., Favier A., Guillemin C., Ducros V., Arnaud J., Richard M.J. (1992). Selenium, glutathione peroxidase (GSH-Px) and lipid peroxidation products before and after selenium supplementation. Clin. Chim. Acta.

[42] Jochum F., Terwolbeck K., Meinhold H., Behne D., Menzel H., Lombeck I. (1997). Effects of a low selenium state in patients with phenylketonuria. Acta Paediatr..

[43] van Bakel M.M., Printzen G., Wermuth B., Wiesmann U.N. (2000). Antioxidant and thyroid hormone status in selenium-deficient phenylketonuric and hyperphenylalaninemic patients. Am. J. Clin. Nutr..

[44] Rocha J.C., Martins M.J. (2012). Oxidative stress in phenylketonuria: future directions. J. Inherit. Metab. Dis..

[45] Sitta A., Vanzin C.S., Biancini G.B., Manfredini V., de Oliveira A.B., Wayhs C.A., Ribas G.O., Giugliani L., Schwartz I.V., Bohrer D., Garcia S.C., Wajner M., Vargas C.R. (2011). Evidence that l-carnitine and selenium supplementation reduces oxidative stress in phenylketonuric patients. Cell. Mol. Neurobiol..

[46] Calomme M., Vanderpas J., François B., Van Caillie-Bertrand M., Vanovervelt N., Van Hoorebeke C., Vanden Berghe D. (1995). Effects of selenium supplementation on thyroid hormone metabolism in phenylketonuria subjects on a phenylalanine restricted diet. Biol. Trace Elem. Res..

[47] Robert M., Rocha J.C., van Rijn M., Ahring K., Bélanger-Quintana A., MacDonald A., Dokoupil K., Gokmen Ozel H., Lammardo A.M., Goyens P., Feillet F. (2013). Micronutrient status in phenylketonuria. Mol. Genet. Metab..

[48] Singh R.H., Rohr F., Frazier D., Cunningham A., Mofidi S., Ogata B., Splett P.L., Moseley K., Huntington K., Acosta P.B., Vockley J., Van Calcar S.C. (2014). Recommendations for the nutrition management of phenylalanine hydroxylase deficiency. Genet. Med..

[49] McMurry M.P., Chan G.M., Leonard C.O., Ernst S.L. (1992). Bone mineral status in children with phenylketonuria—relationship to nutritional intake and phenylalanine control. Am. J. Clin. Nutr..

[50] Hillman L., Schlotzhauer C., Lee D., Grasela J., Witter S., Allen S., Hillman R. (1996). Decreased bone mineralization in children with phenylketonuria under treatment. Eur. J. Pediatr..

[51] MacDonald A., Lee P., Davies P., Daly A., Lilburn M., Gokmen Ozel H., Preece M.A., Hendriksz C., Chakrapani A. (2008). Long-term compliance with a novel vitamin and mineral supplement in older people with PKU. J. Inherit. Metab. Dis..

